# A Genome-Wide Association Study of Prostate Cancer Susceptibility Using Occupational and Environmental Factors as Confounding Factors

**DOI:** 10.7759/cureus.52926

**Published:** 2024-01-25

**Authors:** Takumi Takeuchi, Mami Hattori-Kato, Yumiko Okuno, Akira Nomiya, Hiroshi Fukuhara, Masayoshi Zaitsu, Takeshi Azuma

**Affiliations:** 1 Department of Urology, Japan Organization of Occupational Health and Safety, Kanto Rosai Hospital, Kawasaki, JPN; 2 Department of Urology, Kyorin University Faculty of Medicine, Tokyo, JPN; 3 Center for Research of the Aging Workforce, University of Occupational and Environmental Health, Kitakyushu, JPN; 4 Department of Urology, Tokyo Metropolitan Tama Medical Center, Tokyo, JPN

**Keywords:** snp, industry, occupation, genome-wide, prostate cancer

## Abstract

Background

In addition to genetic predisposition, occupational and environmental factors are important for the risk of prostate cancer. We investigated the effect of single nucleotide polymorphisms (SNPs) on the development of prostate cancer in Japan, including occupational and industrial history as confounding factors in addition to age, smoking, and alcohol drinking.

Methods

We enrolled 210 prostate cancer patients and 504 male control patients. We conducted four genome-wide association study (GWAS) patterns for prostate cancer development. In the association test, logistic regression models incorporated age, smoking history, alcohol consumption history, and each pattern of industrial/occupational classification.

Results

No SNPs satisfying the genome-wide significance level of 5×10^-8^ were detected in GWAS. SNPs with a suggestive association level of 1×10^-6^ were found near the long intergenic non-protein coding RNA 1824 (*LINC01824*) and tripartite motif family like 2 (*TRIML2*) genes in the GWAS using occupational history as a confounder and near the ribosomal protein S2 pseudogene 25 (*RPS2P25*) gene in the GWAS using industrial history as a confounder. No SNPs that met the suggestive association level were observed in the GWAS that did not include occupational and industrial history.

Conclusion

By adding occupational and industrial history to the confounding factors, there were SNPs detected in the GWAS for prostate cancer development. The consideration of occupational and industrial history may increase the usefulness of GWAS.

## Introduction

There were approximately 288,300 new cases of prostate cancer and approximately 34,700 deaths from prostate cancer in the United States in 2023. Since 2014, the incidence rate has increased by 3% per year overall and by about 5% per year for advanced-stage prostate cancer [1]. In Japan, 92,021 males were diagnosed with prostate cancer in 2018, and there were 12,759 deaths from prostate cancer in 2020 [2].

In addition to genetic predisposition, occupational and environmental factors are important for the risk of prostate cancer. Smoking increases the risk of death from prostate cancer [3], but the overall association with prostate cancer incidence remains unclear [4]. Alcohol drinking is a risk factor for the development of prostate cancer [5-7]. Regarding occupation, being a white-collar worker [8] or a professional in a white-collar industry [9] was reported to be risk factors for prostate cancer. In a meta-analysis, exposure to pesticides and chromium and shift work were risk factors for prostate cancer [10].

In this study, we investigated the effect of single nucleotide polymorphisms (SNPs) on the development of prostate cancer in Japan, including occupational and industrial history as confounding factors in addition to age, smoking, and alcohol drinking. As occupational history is a risk factor for prostate cancer, we added it to the confounding factors in order to detect the genetic contribution more clearly.

A genome-wide association study (GWAS) adopted in this study is a comprehensive genome-wide polymorphism search in order to detect SNPs that are susceptible to a disease or condition. Analyzing the genomes of prostate cancer patients, 1,534 associations were identified, leading to 74 articles as shown in the GWAS Catalog [11].

## Materials and methods

The genome purification was performed using ethylenediaminetetraacetic acid (EDTA)-containing blood (10 mL) collected from 210 prostate cancer patients and 504 male control (non-prostate cancer) patients at the Japan Organization of Occupational Health and Safety, Kanto Rosai Hospital. No special inclusion and exclusion criteria were established for male control patients.

We obtained occupational and environmental data from the Inpatient Clinico-Occupational Database of Rosai Hospital Group (ICOD-R) [12] including occupational history and information on smoking and alcohol drinking, with the provision by the Japan Organization of Occupational Health and Safety. Detailed coding of occupational and industrial history can be found elsewhere [13,14]. We obtained other clinical data from electronic medical records. There were missing values because of the omission or lack of description by patients.

Zaitsu classification of industry/occupation

Zaitsu et al. [9] created a new taxonomy (tentatively named the Zaitsu classification) that combines the classifications of industrial and occupational history to create 12 different categories [15,16].

Clinical and environmental factors

Categorical variables were analyzed using Fisher's exact test between two and multiple groups, and age was analyzed using an unpaired t-test.

Genotyping

The genotyping of samples, quality control of samples, quality control of genotypes, and SNP imputation were previously described [16].

GWAS

We conducted four GWAS patterns for prostate cancer development by logistic linear models with PLINK 2.0, using SNP dosage obtained by SNP imputation with a minor allele frequency (MAF) of >0.01. In the association test, age, alcohol drinking history (yes/no), smoking history (the Brinkman index, ordered category with 0-3 levels), and each pattern of industrial/occupational classifications were added to logistic regression models. We tested four industrial and occupational classifications: (i) one variable with 20 levels for industrial classification divisions, (ii) one variable with 12 levels for occupational classification major groups, (iii) the Zaitsu classification, and (iv) GWAS without occupational and industrial history. The genome-wide significance level at p=5×10^-8^ and suggestive association level at p=1×10^-6^ were used in this study.

## Results

Clinical and environmental factors

The prostate cancer patients were older than the control patients (Table 1). Malignant tumors other than prostate cancer, mainly urothelial cancer, were observed in 28.6% of the prostate cancer group and 70.8% of the control group.

**Table 1:** Clinical and environmental factors Ages were analyzed using the unpaired t-test, whereas the Brinkman index and alcohol drinking were analyzed using Fisher's exact test between two or multiple groups

	Prostate cancer	Control
Age (years)	n=210	n=501
	77.5±7.7	69.9±11.7
P-value	>0.0001	
Brinkman index	n=172	n=420
0	30.80%	26.9%
1	13.4%	17.6%
2	22.7%	27.9%
3	33.1%	27.6%
P-value for 2×4 columns	0.2133	
Alcohol drinking	n=166	n=420
0	21.70%	21.2%
1	78.3%	78.8%
P-value	0.9111	

In this study, a high Brinkman index was classified into four stages, and alcohol drinking history was not different between the prostate cancer cases and controls (Table 1). The distributions of the divisions of industrial classification (Table 2), occupational classification major groups (Table 3), and categories in the Zaitsu classification (Table 4) were not significantly different between prostate cancer cases and controls as a whole. Individually, prostate cancer was less frequent in industrial classification division l (scientific research, professional, and technical services) as shown in Table 2. Prostate cancer was significantly less common (Table 3) in the occupational classification major group f (security workers).

**Table 2:** Distribution of industrial classification divisions in the Japan Standard Industrial Classification (Rev. 13 October 2013) The p-value analyzed among multiple groups using Fisher's exact test was 0.2133 *A p-value of <0.05 in individual 2×2 groups (Fisher's exact test) a, Agriculture and forestry; b, fisheries; c, mining and quarrying of stone and gravel; d, construction; e, manufacturing; f, electricity, gas, heat supply, and water; g, information and communications; h, transport and postal services; i, wholesale and retail trade; j, finance and insurance; k, real estate and goods rental and leasing; l, scientific research, professional, and technical services; m, accommodation, eating, and drinking services; n, living-related and personal services and amusement services; o, education and learning support; p, medical, health care, and welfare; q, compound services; r, services, NEC; s, government, except elsewhere classified; and t, industries unable to classify NEC: not elsewhere classified

Industrial classification	a	b	c	d	e	f	g	h	i	j	k	l	m	n	o	p	q	r	s	t
Prostate cancer (%) (n=152)	2.0	0.0	0.0	11.2	28.9	2.0	2.0	10.5	12.5	3.3	2.0	1.3*	4.6	1.3	2.0	2.0	0.7	1.3	2.0	10.5
Control (n=371)	1.1	0.0	0.0	11.3	24.8	1.6	4.0	8.4	10.0	3.0	1.6	5.9*	3.2	1.9	1.6	2.2	0.5	4.3	4.3	10.2

**Table 3:** Distribution of occupational classification major groups by the Japan Standard Occupational Classification (Rev. 5 December 2009) The p-value analyzed among multiple groups using Fisher's exact test was 0.6252 *A p-value of <0.05 in individual 2×2 groups (Fisher's exact test) a, Administrative and managerial workers; b, professional and engineering workers; c, clerical workers; d, sales workers; e, service workers; f, security workers; g, agriculture/forestry/fishery workers; h, manufacturing process workers; i, transport and machine operation workers; j, construction and mining workers; k, carrying-/cleaning-/packaging-related workers; l, workers not classified by occupation

Occupational classification	a	b	c	d	e	f	g	h	i	j	k	l
Prostate cancer (%) (n=153)	5.2	15.0	15.0	19.0	5.9	0.0*	2.0	12.4	5.9	7.2	2.0	10.5
Control (n=371)	4.3	20.5	15.6	14.3	4.9	2.7*	1.6	12.7	4.9	6.2	2.2	10.2

**Table 4:** Distribution of groups in the Zaitsu classification The P-value analyzed among multiple groups using Fisher's exact test was 0.1564

	Blue-collar industry				Service industry				White-collar industry			
Zaitsu classification	Blue-collar worker	Manager	Professional	Service worker	Blue-collar worker	Manager	Professional	Service worker	Blue-collar worker	Manager	Professional	Service worker
Prostate cancer (%) (n=137)	23.4	4.4	10.2	22.6	1.5	0.7	0.7	20.4	0.0	0.7	6.6	8.8
Control (n=330)	18.8	3.3	10.9	18.2	3.9	2.1	0.9	15.5	2.7	0.3	12.7	10.6

Results of GWAS

**Figure 1:**
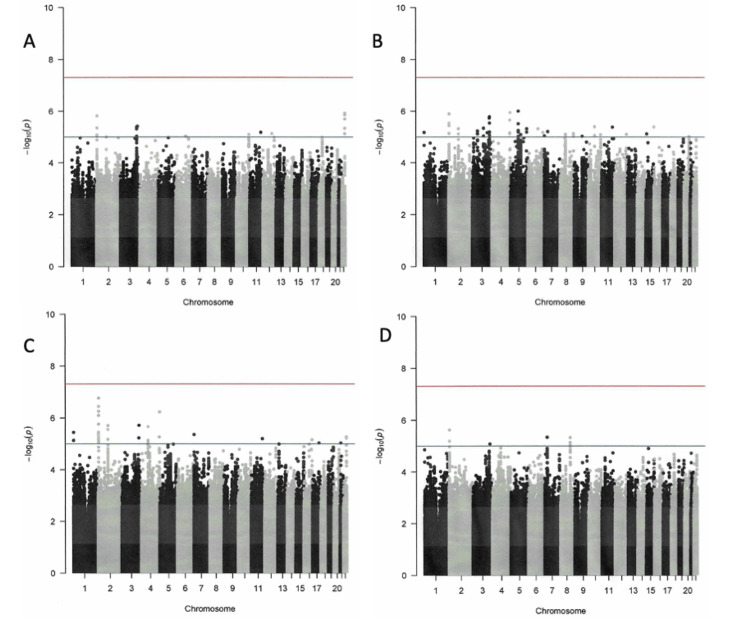
Manhattan plots of the GWAS of prostate cancer cases Industrial/occupational factors added in GWAS: (A) GWAS without industrial/occupational factors, (B) one variable with 20 levels for industrial classification divisions, (C) one variable with 12 levels for occupational classification major groups, and (D) the Zaitsu classification GWAS: genome-wide association study

**Figure 2:**
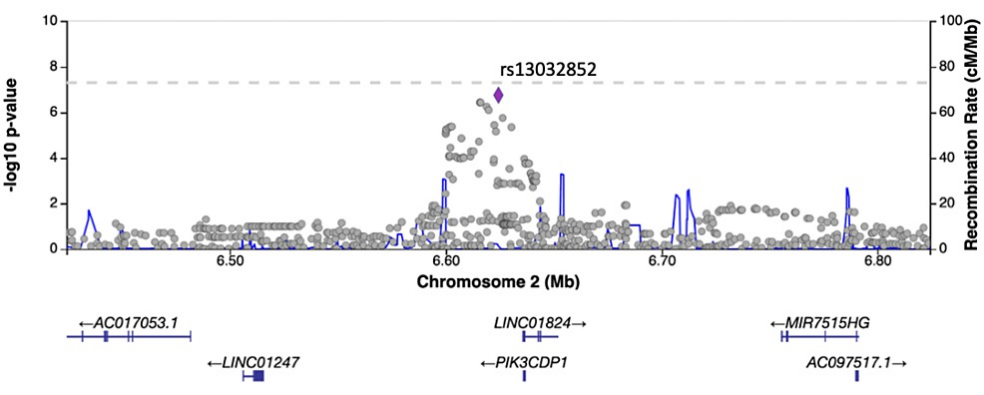
Regional plot of the LINC01824 region (produced by LocusZoom, University of Michigan, Ann Arbor, MI) The added industrial/occupational factor was one variable with 12 levels for occupational classification major groups *LINC01824*: long intergenic non-protein coding RNA 1824

**Figure 3:**
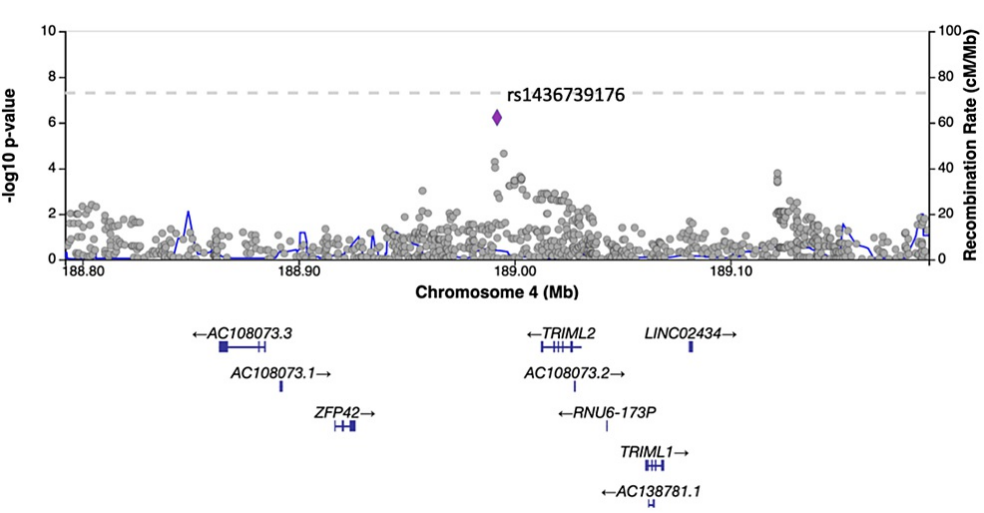
Regional plot of the TRIML2 region (produced by LocusZoom) The added industrial/occupational factor was one variable with 12 levels for occupational classification major groups *TRIML2*: tripartite motif family like 2

**Figure 4:**
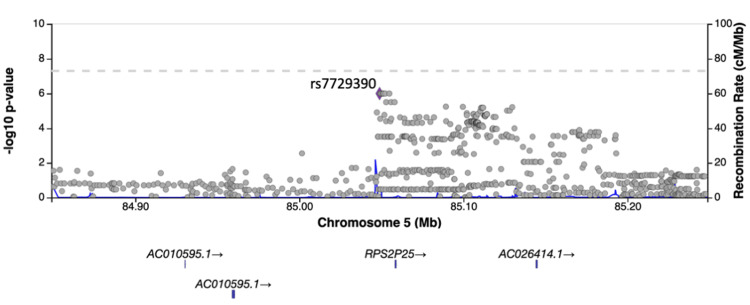
Regional plot of the RPS2P25 region (produced by LocusZoom) The added industrial/occupational factor was one variable with 20 levels for industrial classification divisions *RPS2P25*: ribosomal protein S2 pseudogene 25

No SNPs satisfying the genome-wide significance level of 5×10^-8^ were detected in GWAS. SNPs with a suggestive association level of 1×10^-6^ were found near the long intergenic non-protein coding RNA 1824 (*LINC01824*) and tripartite motif family like 2 (*TRIML2*) genes in the GWAS using occupational history as a confounder (Figures 1-3) and near the ribosomal protein S2 pseudogene 25 (*RPS2P25*) gene in the GWAS using industrial history as a confounder (Figures 1, 4). No SNPs that met the suggestive association level were observed in the GWAS with the Zaitsu classification as a confounding factor or in the GWAS that did not include occupational or industrial history (Figure 1). SNPs that satisfied the suggestive association level of 1×10^-6^ in GWAS are summarized in Table 5.

**Table 5:** Results of GWAS for prostate cancer Industrial/occupational factors added in GWAS: industrial, one variable with 20 levels for industrial classification divisions; occupational, one variable with 12 levels for occupational classification major groups Results that satisfied p<1×10^-6^ by GWAS were selected Chr, chromosome; BP, base pair position; Ref, reference allele; Alt, alternative allele; OR, odds ratio; CI, confidence interval; GWAS, genome-wide association study; *LINC01824*, long intergenic non-protein coding RNA 1824; *TRIML2*, tripartite motif family like 2; *RPS2P25*, ribosomal protein S2 pseudogene 25

Confounding	Chr	rsID	BP	Ref	Alt	OR (95% CI)	P-value	Nearest gene
Occupational	2	rs4669073	6615778	G	A	0.38 (0.27-0.56)	3.66E-07	LINC01824
Occupational	2	rs9678558	6615953	G	C	0.38 (0.27-0.56)	3.66E-07	LINC01824
Occupational	2	rs9678581	6616139	G	A	0.38 (0.27-0.56)	3.66E-07	LINC01824
Occupational	2	rs13015693	6618941	C	T	0.40 (0.28-0.57)	5.57E-07	LINC01824
Occupational	2	rs10929440	6619904	C	T	0.40 (0.28-0.57)	7.96E-07	LINC01824
Occupational	2	rs13032852	6624403	T	G	0.38 (0.26-0.54)	1.75E-07	LINC01824
Occupational	4	rs1436739176	188991855	GA	G	0.41 (0.29-0.59)	5.93E-07	TRIML2
Industrial	5	rs7707213	85048818	T	A	0.45 (0.32-0.62)	9.95E-07	RPS2P25
Industrial	5	rs7729390	85049109	G	A	0.45 (0.32-0.62)	9.95E-07	RPS2P25
Industrial	5	rs7729193	85049256	A	G	0.45 (0.32-0.62)	9.95E-07	RPS2P25
Industrial	5	rs6891075	85049839	C	T	0.45 (0.32-0.62)	9.95E-07	RPS2P25
Industrial	5	rs6895497	85049974	C	T	0.45 (0.32-0.62)	9.95E-07	RPS2P25
Industrial	5	rs17285328	85050882	T	C	0.45 (0.32-0.62)	9.95E-07	RPS2P25
Industrial	5	rs62363123	85051118	T	G	0.45 (0.32-0.62)	9.95E-07	RPS2P25
Industrial	5	rs6861251	85051268	C	T	0.45 (0.32-0.62)	9.95E-07	RPS2P25
Industrial	5	rs9293438	85052413	G	C	0.45 (0.32-0.62)	9.95E-07	RPS2P25
Industrial	5	rs2052900	85054877	G	A	0.45 (0.32-0.62)	9.95E-07	RPS2P25

## Discussion

Kanto Rosai Hospital is located in Kawasaki City, which is a heavy industrial area traditionally neighboring the Tokyo metropolitan area. Accordingly, there were a limited number of workers engaged in the primary sector of industry (farming, logging, fishing, and forestry) and the mining industry in this study.

This study primarily employed a relatively broad classification of industries/occupations. Thus, the aim of the present study was not to explore the connection between SNPs and particular environmentally exposed substances but rather to analyze the influence of wider industrial/occupational environmental factors, for example, stress stimulation and work environment, as confounding elements in prostate cancer development. Under these conditions, several SNPs were detected near genes (*LINC01824*, *TRIML2*, and *RPS2P25*) by GWAS that may be associated with the development of prostate cancer.

The association between these three genes and prostate cancer has not been previously reported, even in the GWAS Catalog. *LINC01824* is an RNA gene located on 2p25.2 and is affiliated with the long non-coding RNA (lncRNA) class. *LINC01824* is a lncRNA that is strongly correlated with transforming growth factor beta 1 (TGF-β1) expression in triple-negative breast cancer tissue [17]. As TGF-β1 signaling is involved in the tumorigenesis of prostate cancer [18], *LINC01824* can also be involved in prostate cancer development. The *TRIML2* gene, located on 4q35.2, encodes a member of the tripartite motif family of proteins. This protein may be regulated by tumor suppressor p53 and may regulate p53 through the enhancement of p53 SUMOylation [19]. p53 is deeply involved in various aspects of prostate cancer [20]. *RPS2P25* is a pseudogene located on 5q14.3 [21]. For these genes, future clinical and biological studies will be necessary.

Utilizing ICOD-R occupational classification major groups, a relationship between occupations that harbor high physical activity and the reduction of cancer risk was demonstrated [12]. By comparing the categories included in the manufacturing industry division of ICOD-R, it was shown that ureter cancer incidence in workers engaged in electronics is higher than that in workers in food manufacturing [22]. Therefore, it is justified to add the industrial/occupational classification to the confounding factors of GWAS for the examination of the development of cancer. It may emphasize genetic contribution by reducing the contribution of environmental factors. In fact, GWAS performed without occupation and industrial history did not find any SNPs that met the suggestive association level; however, when they were added as confounders, several SNPs were detected in this study.

Several malignant tumor diseases other than prostate cancer were included in the control group. The rationale for the inclusion of many other cancers in the control group of GWAS for prostate cancer may be controversial. Pathways shared by various malignancies are less likely to be detected; however, it can be more effective for pathways specific to prostate cancer to appear.

The limitations of this study are that, compared to the GWAS conducted in recent years by a huge number of cases, the number of cases in this study is by far the smallest. Therefore, it is assumed that only limited results will be obtained from the GWAS itself. However, the genome-wide polymorphism information that accompanies detailed occupational and industrial history is scarce and has an advantage there.

## Conclusions

By adding occupational and industrial history to the confounding factors, SNPs were detected in the GWAS for prostate cancer development. Our findings suggest that including occupational and industrial history increases the usefulness of GWAS.
